# Rheological Properties of Aqueous Sodium Alginate Slurries for LTO Battery Electrodes

**DOI:** 10.3390/polym13203582

**Published:** 2021-10-17

**Authors:** Christina Toigo, Milan Kracalik, Elke Bradt, Karl-Heinz Pettinger, Catia Arbizzani

**Affiliations:** 1 Department of Chemistry Giacomo Ciamician, Alma Mater Studiorum Universitá di Bologna, 40126 Bologna, Italy; christina.toigo2@unibo.it; 2 Institute for Polymer Science, Johannes Kepler University Linz, 4040 Linz, Austria; milan.kracalik@jku.at (M.K.); elke.bradt@jku.at (E.B.); 3 Technology Center for Energy, University of Applied Sciences Landshut, 94099 Ruhstorf, Germany; karl-heinz.pettinger@haw-landshut.de

**Keywords:** lithium-ion battery, sodium alginate, biopolymer, LTO, rheology, flow behaviour

## Abstract

Rheological properties of electrode slurries have been intensively studied for manifold different combinations of active materials and binders. Standardly, solvent-based systems are under use, but a trend towards water-based electrode manufacturing is becoming more and more important. The different solvent is beneficial in terms of sustainability and process safety but is also accompanied by some disadvantages such as extraction of residual humidity and a higher complexity concerning slurry stability. Li_4_Ti_5_O_12_ (LTO) active material provides good long-term stability and can be processed in aqueous solutions. Combining the LTO active material with sodium alginate (SA) as a promising biobased polymer binder reveals good electrochemical properties but suffers from bad slurry stability. In this work, we present a comprehensive rheological study on material interactions in anode slurries consisting of LTO and SA, based on a complex interaction of differentially sized materials. The use of two different surfactants—namely, an anionic and non-ionic one, to enhance slurry stability, compared with surfactant-free slurry.

## 1. Introduction

Intensive research on the optimisation of lithium-ion batteries (LIBs) is currently underway due to the ongoing decarbonisation of the economy and rising demand for energy storage systems. Great amounts of energy have to be stored for electric vehicles, smart homes, and manifold further applications. The commercially used active materials on the anodic side of LIBs are limited to a rather small number—namely, graphite, lithium titanium oxide, or silicon-based materials [1]. All of these active materials have their pros and cons; for example, graphite, as the most used material, exhibits the formation of an unstable solid electrolyte interphase (SEI). In combination with the fact that safety concerns such as lithium plating and the formation of lithium dendrites are to be addressed, the long-term operation reliability of graphite anodes is at least questionable [2]. Silicon is a further possible anode material with a high gravimetric capacity of 3600 mAh g^−1^ and several advantages such as low toxicity and high natural abundance [3]. Nevertheless, it suffers from both low conductivity and low initial Coulombic efficiency [4]. The weightiest disadvantage is the large volume change that leads to anode self-pulverisation [5] during several cycles of charging and discharging. Spinel-type lithium titanium oxide (Li_4_Ti_5_O_12_, LTO) is another attractive anode material having a good C-rate capability, fast lithium intercalation, and high cycling stability [6]. Its low electronic conductivity and poor Li^+^ diffusion coefficient result in a low theoretical capacity of 175 mAh g^−1^ [7]. Only a few studies cover the topic of environmentally friendly, water-based preparation of LTO battery slurries using different binder systems without organic solvents. In previous studies, we examined the combination of LTO with sodium alginate (SA) as a binder, resulting in good cycling stability up to 5C but also found out that the combination showed a quick de-mixing of the materials in slurry [8].

The replacement of synthetic binders such as PVDF or PTFE by natural polymers showing properties such as sustainability, biodegradability, and low or no toxicity, will be a major futural task for technical applications.

SA is a linear copolymer composed of β-D-mannuronic acid and α-L-guluronic acid monomers linked by a β-(1-4) glycosidic bond [9]. It is usually extracted in form of sodium salt from brown algae and used for manifold applications in textile, cosmetical, food, and biomedical surroundings. Its good gelling ability, stabilising properties, and high viscosity in water make it an attractive candidate for a high variety of applications [10].

**Figure 1:**
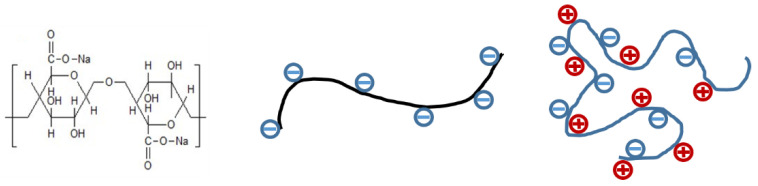
Scheme of SA (**left**) and its polyelectrolyte structure: (**middle**)—rigid chain in a salt-free environment; (**right**)—random-coil chain in a salt-containing environment.

Sodium alginate is a typical polyelectrolyte; it contains negative charges on its backbone which strongly influence its rheological behaviour in solution [11]. The so-called ‘polyelectrolyte effect’ is known to cause the typical upward bending of reduced viscosity versus concentration plot by intra-chain electrostatic repulsion of charges [12]. Several other properties, such as spinnability, are negatively influenced by this effect and have been tried to overcome by the addition of Ca^2+^ cations [13]. It was assumed that chain entanglement as intermolecular interaction could be improved by hydrogen bonds or electrostatic forces [13]. Typically, the SA polyelectrolyte dissociates in an aqueous medium to form an anionic polymer. The rather rigid chain—caused by the repulsion of negatively charged groups—is entangled with an increasing salt concentration in solution, as shown in Figure 1.

Several research groups have evaluated the rheological properties of SA [11,14,15], some with respect to its spinnability [9,13,16]. Rheological characteristics of LIB slurries can be found more frequently [17,18,19,20], but none of them deals with SA in combination with lithium titanium oxide (LTO) as anode material. Both Garcia et al. [21] and Cuesta et al. [22] studied alginate suspensions as binders for LIBs but used graphite as electrode material. Phanikumar et al. [23] investigated SA and polyvinyl alcohol as aqueous-based binders for LTO anodes but did not present rheological properties [23]. As mentioned above, with an increasing demand for LIBs, cell chemistries besides graphite on the anodic side are of strong interest for futural developments.

**Figure 2:**
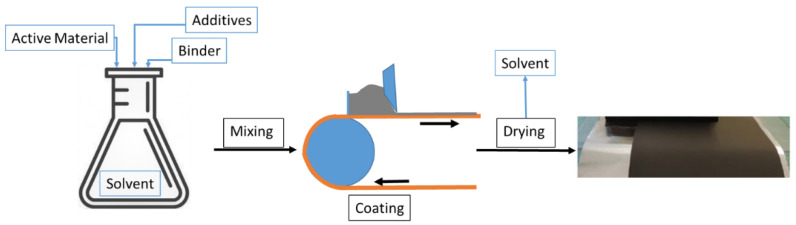
Scheme of the electrode production process.

The preparation of battery slurries is a challenging topic in which a huge variety of different processes are combined and need to be coordinated. One crucial property of a slurry is its optimum rheological behaviour for the casting process onto the current collector [21]. The manufacturing process for battery electrodes is somehow standardised but can of course vary for different application methods. The basic process is described in Figure 2.

The single components such as active material, binder(s), and conducting additive(s) are mixed in a solvent, following defined, sequential steps in which the rheological properties of the slurry play a prominent role. The choice of solvent is dependent on factors such as solubility, availability, and costs. The most commonly used solvent is N-methyl pyrrolidone (NMP), which is flammable and is also listed as a toxic substance. Based on these disadvantages, more and more research focuses on the replacement of NMP by the use of water as a solvent, as there would no longer be the need to address the issues of toxicity, flammability, and the design of explosion-proof devices. The result of the mixing process is a slurry which is coated on the current collector—namely, copper or aluminium foil. A property of extreme importance during processing is the maintenance of a stable slurry within time.

In order to gain a deeper understanding, the flow parameters of slurries need to be evaluated and balanced with the desired production method. Optimum slurry viscosity is indispensable for electrode coating by defining the resulting electrochemical performance. A uniform distribution of materials leads to uniform porosity, thus leading to optimised electrolyte penetration which has a decisive influence on electrochemical performance.

In a preceding investigation, we have already reported a de-mixing of LTO-slurries containing SA as a binder or part of the binder system [8]. The topic of slurry stability was also addressed by Bauer et al. [24], who investigated nanoscaled LFP and micron-sized NMP and came to the conclusion that stabilisation of active material particles can only be achieved by a suitable combination of polymeric binder and particulate additives. Obviously, many battery materials are too large to be prevented from settling, even if they are stabilised as individual particles [24]. Ouyang et al. [25] claimed three common strategies to improve the anti-settling stability of the slurry: first, the application of electrostatic effects or spatial barriers to the particles; second, a reduction in the particles’ mobility by increasing the viscosity; third, the formation of a weakly coagulated state among the solid particles in the slurry. Phase separation was also found by Garcia et al. [21], during which agglomerates began to form between carbon black particles and the SA binder. Furthermore, it was also found out that carbon binder from phenolic resin is able to decrease the geometric surface of carbon black particles, as well as the free space of aggregates and agglomerates [26]. A gel-building ability of SA is reported only in the presence of cations, especially Ca^2+^ ions, which, in general, facilitate chain aggregation and gelation [27]. It is known that different additives enable optimisation of application properties of biobased polymers, for example, poly (lactic acid), one of the most promising sustainable alternatives to petroleum-based polymers [28,29].

Looking at highly dispersed systems containing nanoparticles, one can find three-dimensional networks due to interactions between mineral layers and polymer chains which can be investigated by rotational rheometry in order to evaluate melt elasticity [30]. The used LTO particles are in a dimension of 700 to 1600 nm (D_50_) and therefore are no longer ascribed as nanoparticles. Nevertheless, the possibility of network formation can be examined by evaluation of slurry viscosity (indicating shear-thinning behaviour) and storage modulus curves (indicating the formation of secondary plateaus) [30].

To visualise these effects, including the reinforcement level as a result of a three-dimensional network between SA polymer chains and LTO, a calculation of cumulative storage factor (*CSF*), as described by Kracalik [30], was conducted according to Equation (1).

(1) $$CSF=\overset{628\;\mathrm{rad}/s}{\underset{0.1\;\mathrm{rad}/s}{\int }}G^{\prime }/\overset{628\;\mathrm{rad}/s}{\underset{0.1\;\mathrm{rad}/s}{\int }}G^{\prime \prime }$$

Using this novel analytical approach, internal material enforcement derived from internal molecular friction (change in viscosity, known as cumulative complex viscosity, CCV) can be divided from external reinforcement coming from a 3D physical network—defined as *CSF*.

One possible characterisation method for the de-mixing of suspensions is the degree of flocculation, by which the sedimentation of particles is measured. Due to the fact that LTO active material and graphite are of dark grey and black colour, this method could not be applied to our battery slurry. Therefore, we concentrated on both rheological and contact angle measurements.

**Figure 3:**
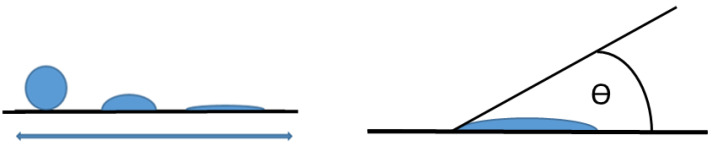
Different droplet forms on a substrate (**left**) and contact angle measurement (**right**).

Contact angle (CA) measurements are the preferred choice to investigate the wettability of surfaces. In general, a contact angle depends on how a liquid forms boundary with the solid states (substrates). This is mainly dependent on the substrate properties such as composition and porosity but also on the liquid’s surface tension. As depicted in Figure 3, several different droplets form on a surface. The droplet on the left, for example, has a very large contact angle, as it does not spread over the surface at all [31], indicating a hydrophobic behaviour. The scheme on the right gives a 2D cross section of a droplet with a marked contact angle.

In order to improve slurry stability, two different surfactants were added to the slurries at a concentration of 0.5%—namely, a polymeric fluorochemical (FC4430, purchased from 3M) and an ammonium salt in water (AA4040, purchased from BASF). The perfluorinated dispersant is known to have good electrochemical stability [32]. The anionic dispersant is also used to improve lithium-ion electrode slurry processing [33].

## 2. Materials and Methods

The SA was a commercially available product from Sigma Aldrich (Taufkirchen, Germany). Furthermore, the LTO slurry formulation consisted of Li_4_Ti_5_O_12_ (GN-LTO-1, GelonLIB, Dongguan, China), particle size D_50_ 0.7–1.6 μm), conductive carbon (Super C65, Imerys, Bodio, Switzerland) and was produced without (Sample SA3) or with (Sample SAD1 and SAD2) 0.5% of detergent. Processing of the electrode slurries was carried out by mixing LTO active material (90%), conductive carbon (6%), and sodium alginate (4%) in deionised water to reach a solids content of 35% in a high-speed dissolver (Dispermat CV3-plus, VMA Getzmann GmbH, Reichshof, Germany). The following commercially available surfactants were used to improve slurry stability and viscosity: FC4430 (3M, Burgkirchen, Germany)—non-ionic, CO_2_-philic dispersant, a combination of 90% polymeric fluorochemical and 8% non-fluorochemical actives in 2% co-solvent (DPM, toluene) and AA4040 (BASF, Ludwigshafen, Germany)—anionic dispersant, with polyacrylic acid and ammonium salt as active ingredients in the water.

Both surfactants were taken from a pre-prepared solution of 10% dissolved in deionised water and diluted further in deionised water to reach a total concentration of 0.5%. The slurries were prepared in the order shown in Table 1.

The rheological measurements have been performed in the forms of a viscosity test, amplitude sweep, and frequency sweep with an MCR 502 Rheometer (Anton Paar, Graz, Austria) using a double-gap geometry (DG26.7). Rheological measurements were taken from slurries 24 h after preparation but with a homogenising step before measurements. Amplitude sweep tests in a range of amplitude γ = 0.001–1% and an angular frequency of 10 rad/s were performed. Frequency sweep tests were conducted using an amplitude γ = 0.01% and an angular frequency Ω = 0.1–628 rad/s. The temperature varied from 20 to 50 °C, with increments of 10 °C.

Contact angle measurements were performed with a CA System OCA from Data Physics Instruments (Filderstadt, Germany). A sample drop of 1 μL was placed on a piece of aluminium foil (sessile-drop method) at room temperature and ambient conditions. It was immediately measured, and a photograph was taken by a camera. The optical measurement was evaluated by the Laplace–Young method. Each slurry was measured three times, and the average was used as the overall resulting contact angle.

A field emission scanning electron microscope (FE-SEM) (Merlin Compact, Zeiss, Germany) with energy-dispersive X-ray spectroscopy (EDX) was used to take EDX element mapping images to check binder distribution.

## 3. Results and Discussion

**Figure 4:**
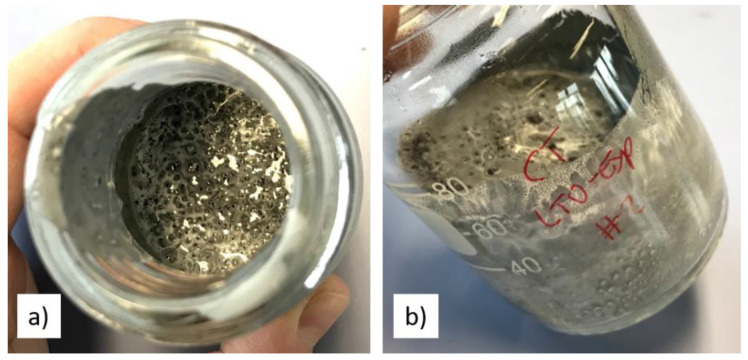
Photograph of de-mixing slurry with small particles of carbon black on the water surface—directly after mixing in top (**a**) and side (**b**) view.

Figure 4 clearly visualises the hydrophobic nature of carbon black, as its particles can be observed on the water surface—directly after and even during the mixing process. This is a known effect for water-based battery slurries [21]. LTO possesses quite low surface energy of less than 2 J/m^2^, indicating very little elastic strain energy associated with coherent interfaces [34]. In contrast, for example, LFP is known to have surface energies of 219 mJ/m^2^ which can lead, in combination with its hydrophilicity, to water capture in its voids and agglomerates, thereby also affecting slurry viscosity [35].

**Figure 5:**
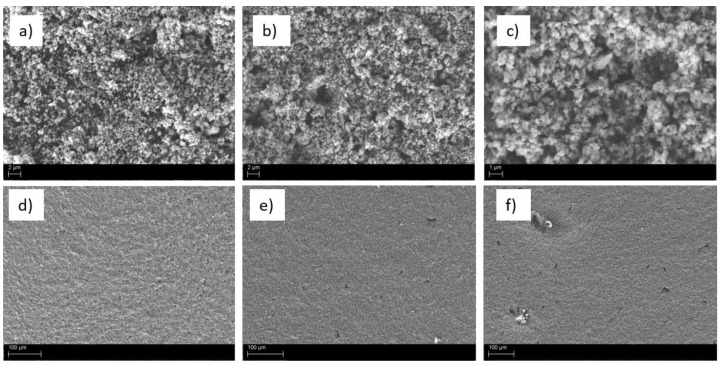
SEM pictures of SA3 (**a**,**d**), SAD1 (**b**,**e**), and SAD2 (**c**,**f**).

SEM images of recipes SA3, SAD1, and SAD2 were collected to evaluate if there is an apparent difference in morphology between the recipe without (SA3) and with the two different dispersants (SAD1 and SAD2), and they are displayed in Figure 5. Detailed images (Figure 5a–c) did not reveal any morphological difference, but a closer look at the overview images (Figure 5d–f) uncovers a difference in coated structure: coated electrodes containing one of the dispersants reveal a clearly smoother and more uniform surface in comparison to the electrode without dispersant.

**Figure 6:**
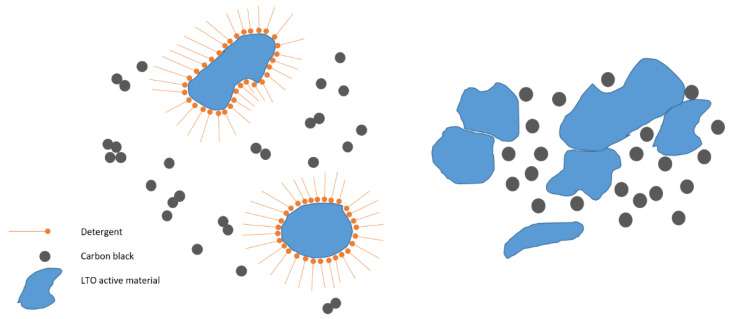
A scheme on detergent influence on active material particles: **left**—with detergent; **right**—without detergent.

A scheme on the general influence of surfactants on active material particles is given in Figure 6, showing the stabilising effect of the surfactant due to particle separation, leading to a lower degree of agglomeration. Due to its bad electric conductivity, LTO implicitly needs a good and uniform carbon black distribution to ensure optimum electrical connection. This can be positively influenced by the use of a surfactant.

Table 2 summarises the detected element mass shares in the investigated electrodes; as presumed, high amounts of titanium, oxygen, and carbon were found in comparable quantities for all three electrodes, contributing around 98% of the detected mass share. Small amounts in the range of 0.39–0.44% of sodium originate from sodium alginate binder, whereas zirconium can be related to impurities.

**Figure 7:**
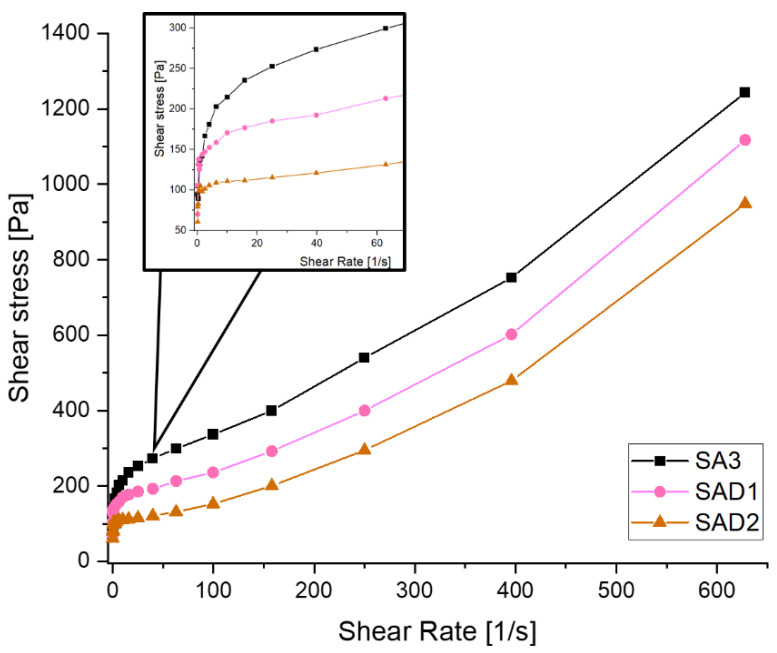
Shear stress vs. shear rate for different SA-LTO slurries.

Figure 7 depicts the three different LTO slurries containing only SA and the two different dispersants in terms of shear rate vs. shear stress. The slurries show dilatant behaviour that increases with shear rate. The addition of a dispersant leads to lower shear stresses, compared with the bare LTO-SA slurry, leading to the suggestion that both dispersants are able to reduce shear stress within the slurries. During the experiment, shear stress increases in the following order SAD2 < SAD1 < SA3.

**Figure 8:**
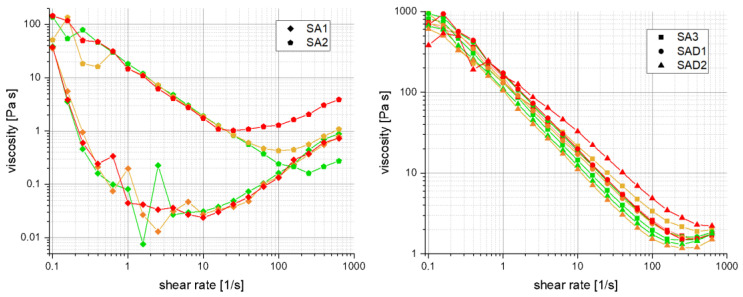
Shear rate vs. viscosity for slurries at different temperatures: 20 °C (green), 30 °C (orange), 40 °C (red). On the **left** are the slurries without LTO, on the **right** the slurries with LTO.

As shown in the double-logarithmic presented in Figure 8, viscosity decreases with increasing shear rate, which is a typical shear-thinning behaviour caused by the disentanglement of polymer chains. At the elevated shear rate, viscosity increases with shear rate—the so-called dilatancy or shear-thickening behaviour caused by the formation of clusters, leading to an increase in viscosity. This behaviour is clearly visible for slurries without active material—namely, mixtures of SA in water (SA1) and SA in water with carbon black (SA2).

What is also evident at first glance is the fact that the addition of carbon black massively influences slurry viscosity. The critical shear rates for the shift between shear-thinning and shear-thickening behaviour are thereby shifted from 8 s^−1^ to 30 s^−1^, depending on temperature. This shift in viscosity also occurs for more complex slurry compositions showing an overall stable behaviour. Compared with the influence of temperature, detergents seem to have a minor influence on slurry viscosity. Obviously, the surfactants do not significantly affect slurry viscosity and show similar results, both in the size of magnitude and pseudoplastic feature.

For frequency sweep measurements, depicted in 9, the storage modulus *G*′ dominates over the loss modulus *G*″, which is a typical behaviour for gel-type systems [24], indicating a three-dimensional network within the slurry mixture. Both *G*′ and *G*″ reveal the highest values for SA3 30 min after preparation being the complete LTO-SA slurry without dispersant. Slurries containing FC4430 as dispersant show the lowest values, both directly after preparation and 30 min later. *G*′ showed rather low slopes up to an angular frequency of about 100 s^−1^, followed by a strongly increasing slope for the samples directly after mixing. This indicates a formation of *G*′ secondary plateau, reflecting a strong physical network in the system, such as the interaction of the LTO particles with SA. This so-called ‘rubber-like behaviour’ indicates to which acting force the physical network/gel structure is stable. Defining a yield point as the crossing of *G*′ and *G*″ curves leads to the assumption that only SAD1 and SAD2 own a yield point at an angular frequency of about 400–500 s^−1^.

In contrast to the massively increasing slope of un-settled slurry mixtures storage modulus at angular frequencies above 100 s^−1^, the slopes of samples containing a dispersant (SAD1 and SAD2) dramatically decrease when settled for 30 min. Due to a maximum measured angular frequency of 628 rad, the behaviour of SA3 sample without dispersant cannot be predicted.

**Figure 9:**
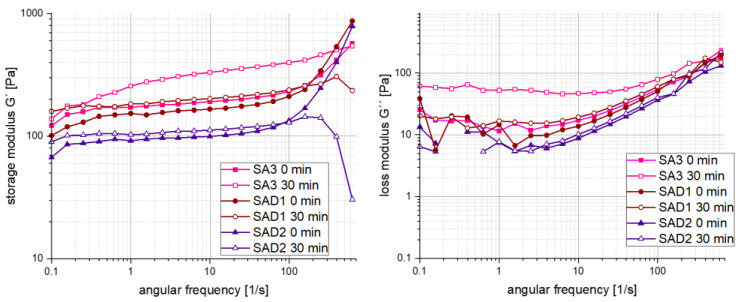
Storage and loss modulus for three different SA-based slurries.

In contrast to Figure 8, a dependency of slurry stability on the use of surfactants is visible in Figure 9, at least for the measurements after 30 min. The decreasing storage factor evinces a decrease in stability at elevated angular frequencies, assuming a non-beneficial surfactant influence. The frequency-dependent modulus indicates that a gel structure in the slurry no longer exists above a critical acting force, demonstrated in this case as a shear rate [20].

**Figure 10:**
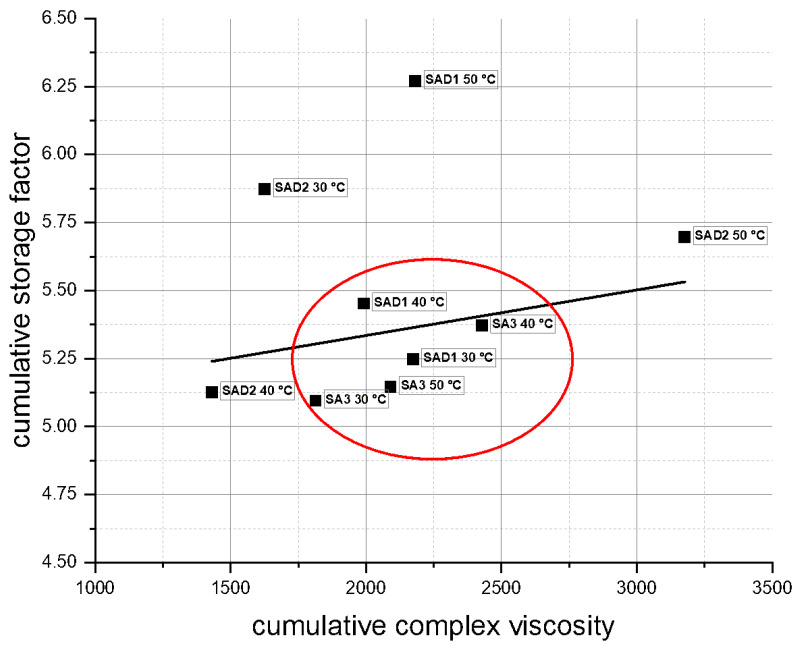
Cumulative complex viscosity vs. cumulative storage factor for all tested slurries.

The results of *CSF* evaluation by integrating over *G*′ and *G*″ according to Equation (1) are shown in Table 3 and visualised in Figure 10.

Plotting *CSF* over CCV shows a stable regime at medium values of 1800–2400 for CCV. In this area, mostly slurries without detergent (SA3) are located, indicating an inverse behaviour of the detergent, thereby showing no stabilising effect. This finding is in accordance with storage and loss modulus evaluation and is also confined by shear rate and shear stress results. It can be clearly seen that the highest material reinforcement occurs for samples SAD1 50 °C and SAD2 30 °C. This can be attributed to an uneven surfactant distribution, combined with a too high concentration.

**Figure 11:**
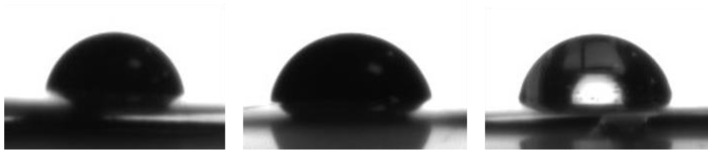
CA measurements, exemplary for SAD2 (54.98°), SAD1 (66.77°), and SA3 (74.17°).

**Figure 12:**
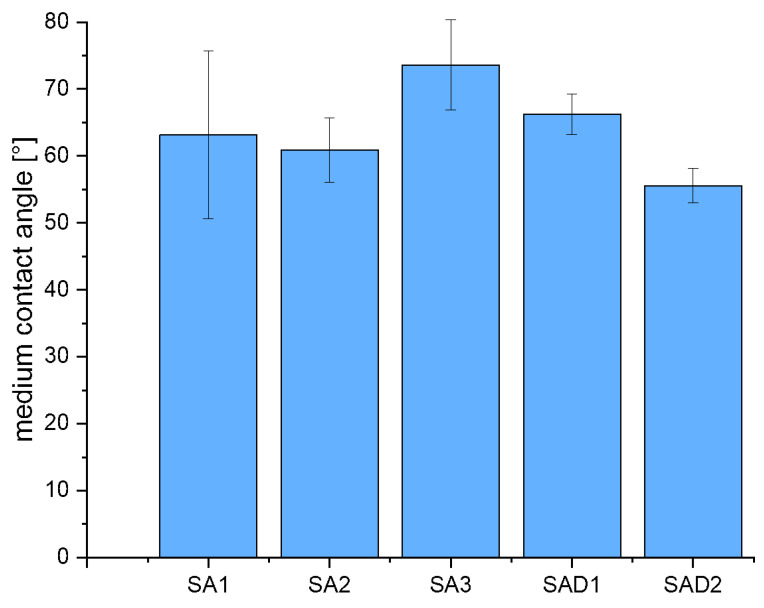
Medium contact angle for different tested slurries, equipped with error bars.

Results of contact angle measurements of mixtures using aluminium foil are outlined in Table 4. It can be seen that both surfactants have an impact by lowering the CA and can thereby improve slurry stability by lowering the viscosity. Figure 11 gives some exemplary measurements for the case of aluminium foil, also indicating a higher contact angle for the LTO-slurry without surfactants. The sharply decreasing standard deviation, as depicted in Figure 12, for slurries containing detergents is noticeable and clearly indicates a stabilisation of slurry properties in general when compared with the slurries without surfactants.

## 4. Conclusions

We investigated aqueous electrode slurries comprising of SA binder and LTO active material with respect to their rheological properties. We demonstrated that tailoring of slurries with the help of dispersants is a practicable way to improve slurry stability and viscosity in general, as confirmed by scanning electron microscopy and CA measurements. Nevertheless, further stabilising effects besides settling prevention are not visible.

This method of surfactant addition can also be of concern for materials with inappropriate rheological properties with respect to various coating techniques. An evaluation of the influence upon electrochemical properties is a further task, which was outside the scope of this study.

## Figures and Tables

**Table 1:** Slurry characteristics and composition.

Description	Recipe Code	Composition [% SA/LTO/cb/Surfactant]
SA-H_2_O	SA1	4/0/0/0
SA-H_2_O-cb	SA2	4/0/6/0
SA-H_2_O-cb-LTO	SA3	4/90/6/0
SA-H_2_O-cb-LTO-Dispex	SAD1	4/90/6/+0.5 Dispex
SA-H_2_O-cb-LTO-FC4430	SAD2	4/90/6/+0.5 FC4430

**Table 2:** Mass shares derived from EDX measurement.

Element	SA3	SAD1	SAD2
	Mass-Share [%]		
C	8.97	7.48	8.74
O	36.11	39.09	39.47
Na	0.38	0.43	0.44
Al	0.09	0.08	0.09
P	0.11	0.12	0.11
K	0.15	0.15	0.07
Ti	52.98	51.4	49.88
V	0	0	0.12
Zr	1.21	1.25	1.1

**Table 3:** Cumulative complex viscosity (CCV) and cumulative storage factor (CSV) for all tested slurries.

Recipe Code and T [°C]	Cumulative Complex Viscosity	Cumulative Storage Factor (*G*′/*G*″)
SA3 30 °C	1814.19	5.095
SA3 40 °C	2428.33	5.372
SA3 50 °C	2091.56	5.146
SAD1 30 °C	2173.85	5.248
SAD1 40 °C	1992.14	5.452
SAD1 50 °C	2182.24	6.270
SAD2 30 °C	1626.29	5.873
SAD2 40 °C	1431.91	5.125
SAD2 50 °C	3176.76	5.696

**Table 4:** Results of CA measurements including standard deviations.

Recipe Code	Medium Contact Angle [°]	Standard Deviation [°]
SA1	63	13
SA2	60	5
SA3	75	7
SAD1	66	3
SAD2	56	3

## Data Availability

The raw data presented in this study are available on request from the corresponding author. The data are not publicly available for the sake of clarity.
